# Evaluation of the equivalence of different intakes of Fruitflow in affecting platelet aggregation and thrombin generation capacity in a randomized, double-blinded pilot study in male subjects

**DOI:** 10.1186/s40795-021-00485-5

**Published:** 2021-12-06

**Authors:** Ranjit K. Das, Tanushree Datta, Dipankar Biswas, Ruedi Duss, Niamh O’Kennedy, Asim K. Duttaroy

**Affiliations:** 1 Department of Nutrition, Institute of Basic Medical Sciences, Faculty of Medicine, University of Oslo, Oslo, Norway; 2 DSM Nutritional Products Ltd, 4002 Basel, Switzerland; 3 Provexis PLC, c/o The University of Aberdeen, Polwarth Building, Foresterhill, Aberdeen, UK

**Keywords:** Fruitflow, Water-soluble tomato extract, Platelet aggregation, Thrombin generation capacity, Equivalence, Integrative medicine

## Abstract

**Background:**

The water-soluble tomato extract, Fruitflow® is a dietary antiplatelet which can be used to lower platelet aggregability in primary preventative settings. We carried out a pilot study to investigate the range of intakes linked to efficacy and to make an initial assessment of variability in response to Fruitflow®.

**Methods:**

Platelet response to adenosine diphosphate (ADP) agonist and thrombin generation capacity were monitored at baseline and 24 h after consuming 0, 30, 75, 150 or 300 mg of Fruitflow® in a randomized, double-blinded crossover study in male subjects 30–65 years of age (*N* = 12). Results were evaluated for equivalence to the standard 150 mg dose.

**Results:**

Results showed that the changes from baseline aggregation and thrombin generation observed after the 75 mg, 150 mg, and 300 mg supplements were equivalent. Aggregation was reduced from baseline by − 12.9 ± 17.7%, − 12.0 ± 13.9% and − 17.7 ± 15.7% respectively, while thrombin generation capacity fell by − 8.6 ± 4.1%, − 9.2 ± 3.1% and − 11.3 ± 2.3% respectively. Effects observed for 0 mg and 30 mg supplements were non-equivalent to 150 mg and not different from baseline (aggregation changed by 3.0 ± 5.0% and − 0.7 ± 10.2% respectively, while thrombin generation changed by 0.8 ± 3.0% and 0.8 ± 3.1% respectively).

**Conclusions:**

The data suggest that the efficacious range for Fruitflow® lies between 75 mg and 300 mg, depending on the individual. It may be pertinent to personalize the daily intake of Fruitflow® depending on individual platelet response.

**Trial registration:**

ISRCTN53447583, 24/02/2021.

## Introduction

Platelets play a key role in blood homeostasis; their responsiveness to a diverse range of external stimuli allows them to respond to vascular stress and helps to preserve the integrity of the vascular system. However, this sensitivity can also result in a hyperaggregable state, in which circulating platelets become chronically sensitised by stress signals from the blood and vascular endothelium, and in these circumstances platelets can also contribute to pathologic processes [1, 2]. Platelet influence on the development and progression of cardiovascular disease (CVD) is well established, especially in atherosclerosis and acute thrombotic events. Individuals with insulin resistance, obesity and diabetes all show characteristics of the hyperaggregable state, which ultimately leads to increased atherosclerotic plaque development, higher blood pressure and high risk of plaque rupture [3, 4]. In individuals at risk of an acute thrombotic event, the benefit of antiplatelet agents such as aspirin, clopidogrel, and glycoprotein IIb/IIIa inhibitors abciximab / eptifibatide is clear.

However, antiplatelet drugs are not suitable for use where risk of a cardiovascular event is relatively low. It is therefore important to find alternative safe antiplatelet inhibitors for the vulnerable population with hyperactive platelets, in order to slow the progression of cardiovascular disease. The number and quality of clinical trials of potentially interesting botanicals and herbal medicines have both increased greatly in recent years, as the fields of complimentary and integrative medicine become better regulated and more established [5]. Many novel therapeutics for treatment of different aspects of the metabolic syndrome, or early stage diabetes – for example regulation of glucose metabolism, or alteration of lipid metabolism – are now suggested [6, 7]. Dietary antiplatelets have also become of greater interest as the range of applications for such interventions becomes more evident in primary prevention. Nutritional / botanical approaches to lowering platelet hyperactivity are attractive, as quite a range of plant-derived antiplatelets exist, although the amount of data available for most remains relatively low as yet [8–10].

We have previously researched the response of platelets to the natural antiplatelet Fruitflow® [11, 12] a water-soluble tomato extract containing a range of antiplatelet compounds and marketed globally as a naturally-derived supplement, with a health claim approved by the European Commission when taken daily at a dosage of 150 mg [13, 14]. From previous studies, a dose of 150 mg Fruitflow® generally causes approximately 15–23% suppression of platelet response to 3 μmol/L ADP agonist. This is a platelet suppression that is not associated with increased bleeding tendencies and thus safe for use by the general population [11].

We know from previous work that as much as a threefold increase in Fruitflow® dosage does not result in a significantly higher effect on platelet aggregation [12], but no data exists on doses lower than 150 mg Fruitflow® per day. Thus, the lower limit of the efficacious dose range is not yet known. In studies in healthy individuals, we have also observed marked variability in the Fruitflow® response range [11–13]. The degree of variability makes it likely that some individuals will be more responsive to Fruitflow than others, and thus may achieve the desired platelet inhibition at lower doses.

Here we describe a pilot study (Clinical trial Registration Number ISRCTN53447583, Date of Registration 24/02/2021) that set out to test different doses of Fruitflow® in a small number of subjects to establish whether doses other than the current standard dose of 150 mg would affect platelet function in a similar manner and to examine the heterogeneity of the response.

## Materials & methods

### Preparation of intervention supplements

Fruitflow® SD is commercially produced by DSM Nutritional Products, Basel, Switzerland, in powder format. The composition of Fruitflow® has been described previously [11]. Briefly, a standard 150 mg dose (as per the approved EC health claim) delivers up to 9 mg nucleoside derivatives, up to 10 mg simple phenolic conjugates (e.g., chlorogenic acid, other caffeic/phenolic acid derivatives), and up to 7 mg flavonoid derivatives, of which at least 2.4 mg are quercetin derivatives. The commercially produced Fruitflow® SD is standardized to ensure minimum quantities of these three compound groups are contained in each manufactured ingredient batch, ensuring that the bioactive components’ intake is consistent from batch to batch. In this study, the powder was administered as a single dose at concentrations of 30 mg, 75 mg, 150 mg, and 300 mg Fruitflow® SD (FF30, FF75, FF150, FF300). Tapioca starch was used as a placebo control (Con) (Essential Nutrition Ltd., Brough, UK). All supplements were encapsulated using size 00 Vegecaps (LGA, La Seyne-sur-Mer, France), and the final weight of each capsule was 600 mg (weight of Fruitflow® SD plus weight of tapioca starch filler).

### Randomization and coding of supplements

Capsules were coded following a randomization protocol: Genstat (VSN International, 17th /18th edition) was used to generate 20 sets of randomly allocated treatments numbered 1–5. Each treatment set was allocated to a subject number, in numerical order, and the appropriate treatments were boxed and labelled with the subject number and visit number, e.g., S1–1, S1–2, S1–3, etc. All supplements were coded off-site at the Human Nutrition Unit of the University of Aberdeen and provided to the investigators prior to the start of recruitment. Subject numbers were then assigned to subjects in order of recruitment into the study. Supplements were identical concerning appearance and only differed in the coding of the capsules. The treatment code of the intervention supplements was blinded for subjects, investigators, and staff involved in the study’s conduct.

### Recruitment and screening of subjects

Fifteen males aged 30–65 years were assessed for eligibility, of which 12 were recruited into the study. Recruitment was carried out from the local population, by poster advertisement within the Faculty of Medicine. Study numbers were estimated on the basis of the expected response to the 150 mg dosage: 15–20% ± 9–15% reduction from baseline ADP-mediated aggregation after 150 mg Fruitflow® SD. For such a response, we calculated a minimum of 10 subjects required to allow the effect to be detected (vs. placebo) with 80% power and a 95% confidence interval for the mean. Extra subjects were recruited to allow for failure to complete all interventions. Suitability for inclusion into the study was assessed using diet and lifestyle questionnaires and medical screening, during which blood pressure and platelet function were evaluated. Individuals with low haematology counts (platelet number < 170 × 10^9^/ L; haematocrit < 40%; haemoglobin < 120 g/L), or low platelet function (as determined by response to 3 μmol/L ADP agonist) were not included into the study. Any subject habitually consuming dietary supplements (for example, fish oils, evening primrose oil) suspended these supplements for a minimum of 1 month before participating in the study.

### Ethical considerations

Written informed consent was obtained from all subjects prior to participation, and all study procedures were in accordance with the Helsinki Declaration of 1975 (revised in 1983). The local ethical committee approved the study at Oslo University Hospital, Norway (Reference No.: 2015/ 396) and it was subsequently registered as ISRCTN53447583.

### Study design

This was an active control equivalence study (a positive control study), following a double-blinded, randomized crossover design, in which the treatment interventions Con, FF30, FF75, and FF300 were compared to FF150 (standard dose). Subjects undertook all five interventions, with each intervention separated by a period of at least 7days. All study activities were undertaken at the Nutrition Dept. of the Faculty of Medicine, University of Oslo, Norway. Each intervention period was of 24-h duration. Subjects presented at the Nutrition Department facility, and baseline measurements, including fasted baseline blood samples (approximately 40 mL), were taken (t0). The intervention supplements, randomly assigned and blinded, were consumed in the presence of study investigators after the baseline venepuncture. Breakfast was then supplied, and subjects were free to leave the facility. After 24 h, subjects returned to the facility, and a 12-h fasted blood sample was taken for analysis (t24). Subjects were again given breakfast and were free to leave the facility, returning after a minimum of 7 days to repeat the procedure for the next interventions, as required, until all five interventions had been completed.

### Study measurements

#### Platelet aggregation assay

For measurement of platelet aggregation at baseline (t0) and post-intervention (t24), blood was mixed with 3·8% trisodium citrate (9:1 (v/v), blood/citrate). Blood collection using the Monovettes system (Sarstedt, UK), platelet-rich plasma (PRP) preparation, and light-transmission aggregometry using an AggRam aggregometer (Helena Biosciences, Sunderland, UK) were carried out as described by us previously [11, 12, 15]. Platelet aggregation in 200 μL adjusted PRP was initiated by the addition of 20 μL of ADP (Helena Laboratories, Beaumont, TX) at concentrations ranging from 1 to 8 μmol/L ADP. Based on the pre-intervention baseline measurements, an ADP concentration was pre-selected for each individual, such that an optimal response could be recorded for each. All measurements were carried out in duplicate, within 2 h of blood sampling. Effects on platelet aggregation observed post-intervention were expressed as the percentage change in the area under the aggregation curve (%AUC) post-intervention compared with baseline values.

#### Thrombin generation capacity (TGC) assay

Thrombin generation capacity (TGC) was measured at baseline (t0) and post-intervention (t24) in citrated platelet-poor plasma (PPP), which was further treated to ensure only microparticles remained in the plasma (no platelets or large platelet fragments). In this way, the TGC related to plasma microparticle load was measured. Aliquots of 25 μL were stored frozen at − 80 °C for up to 1 month before analysis. Analyses were carried out using the Technothrombin® Thrombin Generation Assay (Diapharma Group Inc., West Chester, Ohio, US), using the protocol specified by the manufacturer but with some modification to sample preparation: platelet-poor plasma was generated by double centrifugation directly from citrated whole blood, not sequentially after generation of platelet-rich plasma. The final concentrations of tissue factor (TF) used were 1 pmol/L. Microparticle-free plasma and microparticle-high plasma (Diapharma Group Inc., West Chester, Ohio, US) were used as negative and positive controls.

#### Supplementary measurements

After each blood withdrawal, a haematology analyzer (Hemocytometer, Horiba ABX micros60, Montpellier, France) was used to monitor haematological parameters. Baseline plasma C-reactive protein (CRP) concentration was measured in EDTA-anticoagulated blood using a semi-quantitative latex agglutination assay (Dade Behring, Milton Keynes, UK), which allowed classification of sample CRP status as either ‘normal’ or ‘elevated’ (> 6 ng/ml CRP). Data from samples with elevated CRP was discarded.

### Statistical analysis

Data are presented as mean ± standard deviation (SD). Data from interventions where evidence of platelet pre-activation due to venepuncture existed or where elevated CRP was recorded were removed from the set. Preliminary assessment of the data distribution was carried out by inspecting histograms, and data points classified as outliers were removed. Changes from baseline (t0) within the study population were analysed using a mixed model following the residual maximum likelihood (REML) approach. Initially, random effect terms were subject / (visit x timepoint), while fixed effect terms were (order + treatment) x timepoint. Significance was tested with the Wald statistic. As no significant order x treatment interactions were observed, the model was simplified (without the order term, treatment and visit are equivalents). Random effects then became subject / (treatment x timepoint), and fixed effects were treatment x timepoint. Treatments Con, FF30, FF75 and FF300 were compared to FF150, to determine equivalence or non-equivalence. Due to the small sample size, no post-hoc comparisons were made. Statistical analyses were carried out using Genstat (VSN International, 17th / 18th edition), and differences were considered significant at *P* < 0.05.

## Results

### Subject characteristics

**Fig. 1:**
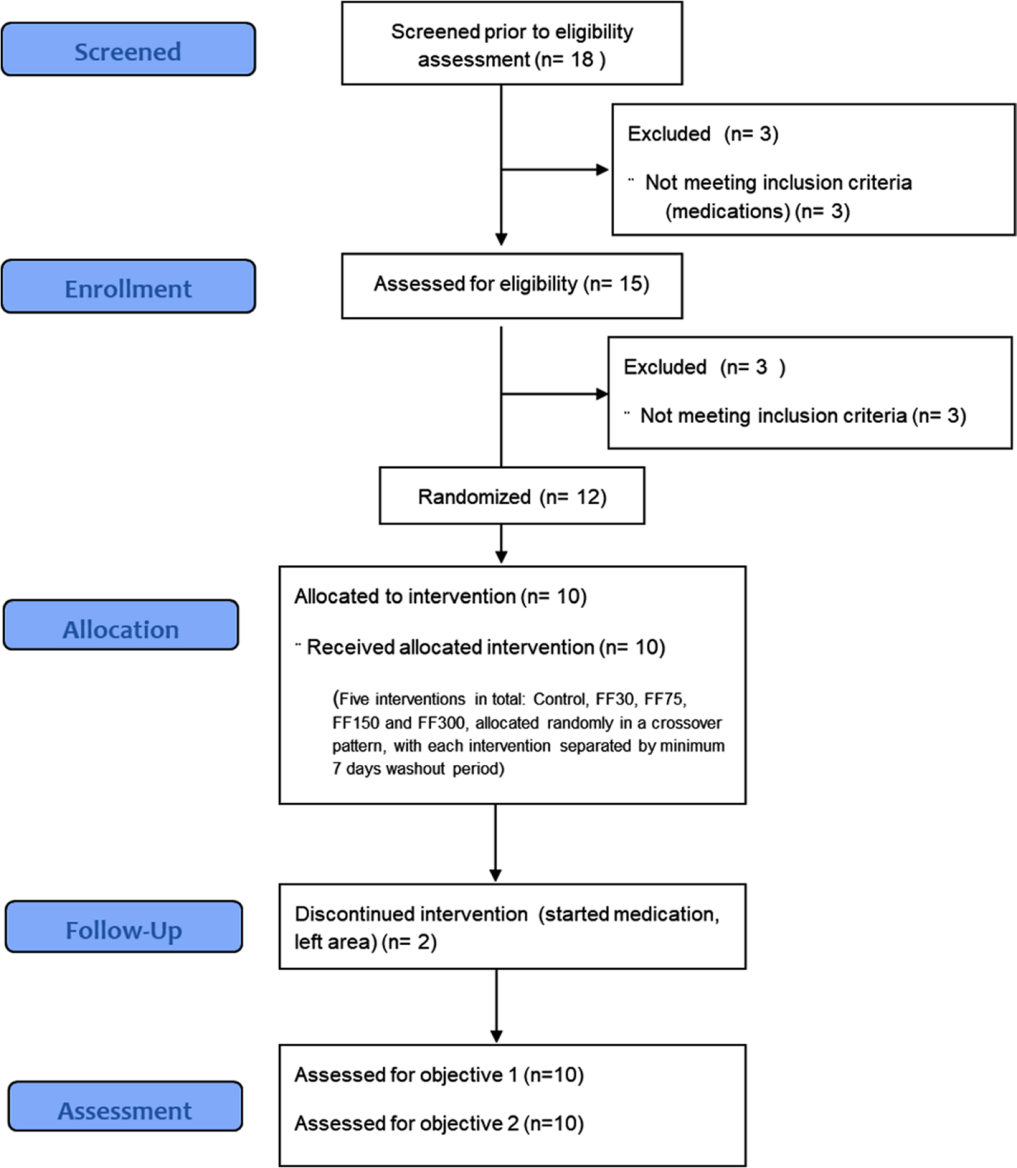
Study participant flow diagram. Flow diagram showing the disposition of subjects throughout the screening, randomization and intervention stages of the study

In all, 12 subjects were recruited into the study, of which ten completed the intervention pattern (see Fig. 1 for a participant flow diagram). The characteristics of the subject group (*n* = 10) at the pre-intervention baseline are shown in Table 1. Subjects were of Asian ethnicity, with one smoker. Eight out of ten subjects were overweight or obese, and only one had a waist/hip ratio < 0.9. Nine out of ten subjects were characterised by hypertension – one with elevated BP (systolic BP 120–130, diastolic BP < 80 mmHg), five with stage 1 hypertension (systolic BP 130–140 and/or systolic BP > 80 mmHg) and two with stage 2 hypertension (BP > 140/90 mmHg). Haemostatic variables were within the normal range and did not differ significantly from the pre-intervention baseline at any of the t0 timepoints. However, the average mean platelet volume (MPV) was relatively elevated, indicating some degree of platelet activation. A wide range of response to ADP agonist was observed, with concentrations ranging from 1 μmol/L to 8 μmol/L generating an optimal response in different individuals. The range observed for TGC was smaller, giving a more homogenous data set. The baseline levels of TGC measured were also relatively elevated, compared to a healthy population. These characteristics generally lead to the conclusion that some degree of platelet hyperaggregability could be expected in this set of subjects.

**Table 1:** Baseline characteristics of the subject group

Parameter	Pre-intervention baseline values
Country of origin	Indian Subcontinent
Age (years)	49 ± 9 (40–65)
BMI (kg/m2)	27.6 ± 3.4 (22.2–34.0)
Smoking status	1 smoker, 9 non-smokers
Resting blood pressure (mm Hg)	134 ± 10 / 85 ± 9 (120/70–150/100)
Waist / hip ratio	1.00 ± 0.00 (0.89–1.04)
Mean platelet volume (fL)	9.0 ± 0.8 (7.6–11.3)
Baseline platelet response to low ADP agonist (%AUC)	77.6 ± 15.1 (48.2–96.0)
Baseline platelet response to high ADP agonist (%AUC)	77.1 ± 10.0 (53.3–96.0)
Thrombin generation capacity (nmol/L)	331 ± 54 (254–420)

Data are shown as mean ± standard deviation, with the range given in brackets. *N* = 12. Low adenosine diphosphate (ADP) agonist concentrations are defined as 1–3 μmol/L. High ADP agonist concentrations are defined as 5–8 μmol/L. Platelet response is measured as % area under the aggregation curve, %AUC

### Intervention with different doses of Fruitflow® affected platelet function and TGC at t24

Table 2 shows the platelet aggregation response to optimal concentrations of ADP agonist in PRP at baseline (t0) and after the intervention (t24), and also sets out the percentage change from t0 after the intervention. Baseline measurements were not significantly different between visits. Treatment effects were observed at t24, with FF75, FF150, and FF300, all significantly reducing the platelet response to ADP, relative to t0, whereas no such effect was observed for Con or FF30. Data for TGC are also shown in Table 2; again, there was no difference between t0 measurements between treatments. TGC was affected similarly to platelet aggregation at t24; TGC was reduced by 15–20% relative to baseline for FF75, FF150, and FF300, but was not significantly reduced for Con or FF30.

**Table 2:** Platelet aggregation response to ADP and thrombin generation capacity at t0 and t24, for each intervention undertaken by the subject group

		Con	FF30	FF75	FF150	FF300
**Platelet aggregation in response to optimal ADP (%AUC)**		*N = 8*	*N = 8*	*N = 10*	*N = 9*	*N = 9*
	t0	72.7 ± 17.6	71.0 ± 12.2	73.6 ± 12.8	74.5 ± 14.9	82.7 ± 13.1
	t24	76.0 ± 15.7	75.7 ± 13.7	64.5 ± 16.3^*^	71.2 ± 17.6^*^	70.3 ± 20.4^*^
	Δ% from t0	3.0 ± 5.0 ^§^	− 0.7 ± 10.2 ^§^	− 12.9 ± 17.7	− 12.0 ± 13.9	− 17.7 ± 15.7
**Thrombin generation capacity (nmol/L)**		*N = 10*	*N = 10*	*N = 10*	*N = 10*	*N = 10*
	t0	324 ± 53	327 ± 59	330 ± 57	333 ± 56	328 ± 59
	t24	327 ± 53	330 ± 62	303 ± 58^*^	303 ± 53^*^	291 ± 54^*^
	Δ% from t0	0 8 ± 3.0^§^	0.8 ± 3.1^§^	−8.6 ± 4.1	− 9.2 ± 3.1	− 11.3 ± 2.3

Data are shown as mean ± standard deviation. Adenosine diphosphate (ADP) agonist concentrations ranged from 1 to 8 μmol/L, and data for t0 and t24 are given as % area under the aggregation curve (%AUC). No significant differences between t0 values were detected between interventions. Where t24 is different from t0, this is indicated with *, while non-equivalence to FF150 is indicated by § (*P* < 0.05)

### Treatment with either FF75, FF150, or FF300 gave equivalent results

Treatment with FF75 or FF300 was equivalent to treatment with FF150, with all interventions reducing platelet response to ADP agonist by 12–18%. Treatments Con and FF30 were non-equivalent to FF150. A similar pattern was observed for TGC, with treatments FF75 and FF300 again showing equivalence to FF150, while treatments Con and FF30 did not.

### Underlying heterogeneity in platelet response to ADP agonist leads to variable responses to Fruitflow® at all doses

**Fig. 2:**
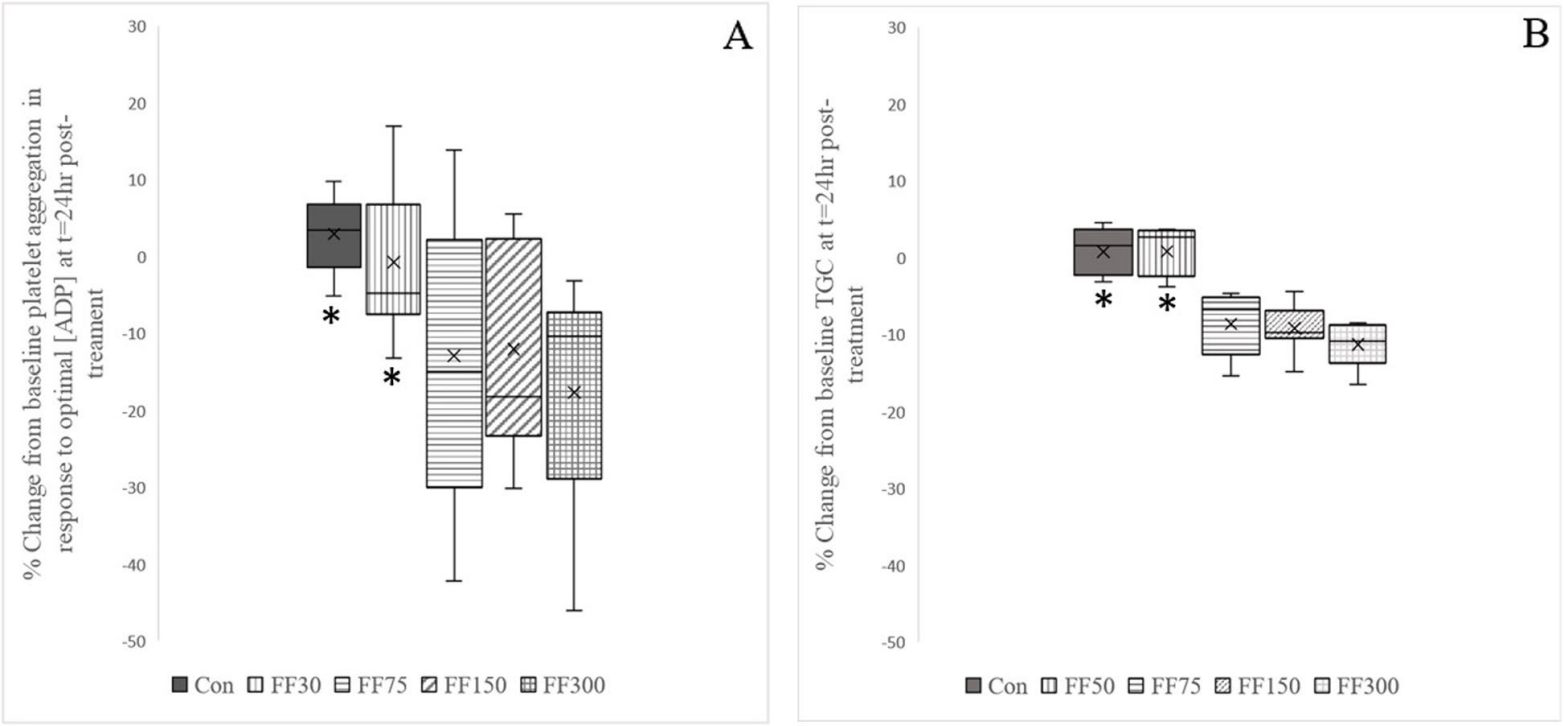
Platelet Aggregation Response. Boxplots constructed to show the range of the % change from baseline recorded for each treatment, for platelet aggregation (maximum % area under the aggregation curve, Max % AUC) at optimal adenosine diphosphate (ADP) concentrations (**A**), and for thrombin generation capacity (TGC) (**B**). ADP agonist concentrations ranged from 1 to 8 μmol/L. In A, treatments Con, FF30, FF75, FF150 and FF300 had *N* = 8, 8, 10, 9 and 9 respectively. In B, N = 10 for all treatments. Median values are shown within the plots as the conventional line. Mean values (as given in Table 2) are shown for convenience, denoted by the marker **x** within each plot. Non-equivalence to FF150 is indicated by * (*P* < 0.5)

The response to Fruitflow® at the different doses examined was variable, as reflected in the changes from baseline aggregation measured at optimal concentrations of ADP (Table 2, Fig. 2).

### TGC followed the same patterns of response to Fruitflow®as platelet aggregation but resulted in a more homogenous data set

Inspection of the TGC data shows a tighter range at the pre-intervention baseline (Table 1), and a smaller SD in the post-intervention responses to treatment (Table 2**,** Fig. 2), compared to the platelet aggregation data at either concentration of ADP. Reductions in TGC for FF75, 150, and FF300 show a trend towards a dose-response, whereas the corresponding aggregation data are too variable for such a trend to be observed (Fig. 2). The two treatments, which are non-equivalent to FF150 (Con and FF30), are also more similar to each other when considering TGC compared to when considering aggregation response, suggesting that there may be a specific reason behind the rise in platelet aggregation observed after intervention with FF30.

## Discussion

Our aims in this pilot study were to examine whether consuming amounts of Fruitflow® less than 150 mg could result in desirable levels of platelet suppression ex vivo, and to assess the platelet response variability to all doses tested. By doing so, we hoped to establish the lower limits of the efficacious intake level, and also to assess the potential value of personalizing the daily intake, based on individual response. The latter aim reflects a recent focus of study on the heterogeneity of platelet response to intervention – demographic and lifestyle-driven differences in platelet behaviour [16–19]. Platelet behaviour varies in response to a wide range of factors – ethnicity, age, nutrition status, exposure to air pollution, exercise levels, etc. [20–29]. No two individuals’ platelets are precisely the same, something that can be clearly observed in the heterogeneity of their responses to agonist and to antiplatelet agents [30–34], Studies using antiplatelet drugs have shown that marked differences in platelet responses may be linked to such factors, with genomic and nutrigenomic reasons postulated [35, 36]. The approach to antiplatelet medication in secondary prevention has begun to evolve, as it becomes clearer that different demographics may respond very differently to fixed doses of antiplatelets [37–39]. This is equally true for dietary antiplatelets, raising the prospect that personalization of the daily intake could be beneficial for optimal effects.

The study was small, and biased, in that all participants were male and from the Indian Subcontinent, and its size necessarily constrained the comparisons between Fruitflow® doses. It was intended to give an indication of the range of intakes displaying efficacy, rather than definitive data. The subjects displayed characteristics consistent with some degree of platelet hyperaggregability, which, although quite typical in the wider population, is a further slight bias.

The platelet aggregation response to ADP agonist was the main outcome measure, and TGC of platelet-depleted plasma was also included as a comparative measure. TGC is a standardized global haemostasis assay that is becoming well established: it incorporates both negative and positive controls [40, 41]. It can be used to examine both thrombin generation kinetics and peak thrombin generation. Generation of thrombin is strongly linked to the concentration of circulating platelet microparticles and thus to platelet hyperaggregability. It is a more flexible method of analysis than platelet aggregometry and more suited to a quick assessment of individual haemostatic status. The ISTH published proposals to standardize sampling and analytical procedures for TGC in 2017 [42], and Dargaud et al. subsequently suggested the use of TGC as a predictive tool to personalize the use of clotting factor replacement in haemophiliacs [43]. TGC was included in this study as an outcome measure to examine whether TGC might be an effective measure of the effects of Fruitflow®.

Our study found that, when compared to the current standard 150 mg daily intake of Fruitflow®, intakes of 75 mg and 300 mg gave equivalent results in terms of both platelet aggregation reduction and TGC reduction, with average ranges of response from 12 to 18% for reduction of ADP-mediated aggregation, and from 15 to 20% for TGC. The control and the 30 mg supplements were not equivalent to the 150 mg dose, with neither causing a significant change from baseline aggregation. Thus, doses of Fruitflow® from 75 mg – 300 mg affected platelet function equivalently in this group of subjects. The observation that 150 mg and 300 mg intakes are equivalent corresponds to findings from our earlier work which also showed that increasing intakes of Fruitflow did not significantly increase the platelet suppression observed, compared to the ‘standard’ intake [12].

The baseline platelet response to ADP agonist was highly variable. Some subjects’ platelets showed 8 times more sensitivity of response to ADP than others, as judged from the agonist concentrations needed to give an optimal aggregation curve (range 1 μmol/L – 8 μmol/L ADP). While this may reflect an inherent heterogeneity in platelet function, it is also possible that lifestyle differences, age differences etc. may have contributed to the variability observed – we did not control or monitor physical activity, sleep patterns or diet, for example. Given the variability in baseline platelet function, it is no surprise that the range of responses to Fruitflow® was similarly heterogeneous, although less so than in other studies. This may have been due to the subject characteristics – the subjects were quite similar in terms of ethnic background and some physical characteristics (e.g., BMI, presence of mild hypertension). Due to the small study size, we could not investigate whether platelets that respond more sensitively to ADP respond more, or less, sensitively to Fruitflow® at this time.

Results obtained using TGC were less variable than those based on aggregometry. They showed a trend towards a dose-response, although the study design did not allow proper analysis of this. Using TGC as a surrogate biomarker for platelet hyperactivity or the platelet response to intervention is an interesting option, especially when the intervention period is less acute – similar to MPV, the full effect of any intervention on TGC is more likely to be observed over a longer timeframe. While TGC in platelet-depleted plasma cannot replace aggregometry in determining the purely platelet-specific response to an intervention, it does provide a linked estimate of microparticle-driven thrombin generation. Data already exists to give normal, low, and elevated TGC ranges in some patient populations, and the method is automatable and has increased flexibility compared to aggregometry.

Despite the limitations of this study, the data generated suggests that that a larger study which addresses the bias contained in this pilot study should be undertaken to confirm that the efficacious dose range for Fruitflow® is between 75 mg and 300 mg; that personalizing the dosage of Fruitflow® could be an option to deliver optimal effects, given the observable heterogeneity of the response between individuals; and besides, that further investigation of TGC as a surrogate marker for monitoring the effects of the intervention on platelet function is warranted.

## Data Availability

Analysed data relevant to the study are included in the article. The datasets generated are not publicly available as set out in agreements with the commercial partners but are available from the corresponding author on reasonable request.
